# Alcohol-Associated Hepatocarcinogenesis: Wnt/β-Catenin in Action

**DOI:** 10.1016/j.ajpath.2025.04.016

**Published:** 2025-05-09

**Authors:** Yuhua Xue, Tian Tian, Melak Ottallah, Mahfuza Mannan, Joshua Barkin, Brady Jin-Smith, Liya Pi

**Affiliations:** Department of Pathology and Laboratory Medicine, Tulane University, New Orleans, Louisiana

## Abstract

Long-term alcohol consumption is a leading global health concern, primarily due to its deleterious effects on liver function and its well-established association with hepatocellular carcinoma. Alcohol-related liver disease (ALD) encompasses a continuum—from reversible hepatic steatosis and steatohepatitis through progressive fibrosis and cirrhosis to overt hepatocellular carcinoma. Accumulating studies have revealed that the Wnt/β-catenin signaling pathway is an essential regulator in ALD pathogenesis, orchestrating diverse molecular, immunologic, and epigenetic processes. Aberrant β-catenin activity disrupts redox homeostasis, promotes chronic inflammation, drives extracellular matrix remodeling, and alters hepatocyte cell fate, thereby creating a microenvironment that is highly conducive to carcinogenesis. This article provides a systematic review of the significant function of Wnt/β-catenin signaling in ALD, emphasizing its regulatory impact on liver fat accumulation, its inflammatory role in steatohepatitis, its involvement in fibrogenesis, and its tumor-promoting effects in alcohol-related hepatocellular carcinoma. In addition, emerging therapeutic strategies that offer potential for early identification and tailored therapy of ALD are explored—including direct Wnt modulators, combinatory therapeutics, and precision medicine approaches.

High levels of alcohol intake continue to pose a significant public health issue worldwide, leading to significant rates of illness and death.^1^ Alcohol-related liver disease (ALD) includes a wide range of liver diseases, starting from basic steatosis and steatohepatitis to more severe conditions such as advanced fibrosis, cirrhosis, and eventually hepatocellular carcinoma (HCC).^2^ The pathogenesis of ALD is multifactorial. Aside from the direct toxic effects of ethanol and its metabolites on liver cells, ALD arises from a complicated interaction of immunologic, metabolic, and epigenetic changes. Chronic oxidative stress, persistent inflammation, and progressive fibrotic remodeling are crucial drivers that disrupt liver homeostasis, compromise hepatic function, and set the stage for malignant transformation.

At the heart of these disease-related mechanisms is the Wnt/β-catenin signaling pathway, a well-preserved regulatory mechanism that plays a pivotal role in modulating cell growth, differentiation, and survival throughout development while sustaining liver homeostasis in adults.^3^ Under normal conditions, β-catenin is sequestered in the cytoplasm by a destruction complex composed of glycogen synthase kinase (GSK)-3β, Axin, adenomatous polyposis coli protein (APC), and casein kinase (CK)-1.^4^ The Wnt ligands initiate the activation of β-catenin signaling by forming the frizzled protein (FZD) and low-density lipoprotein receptor–related protein (LRP)-5/6 receptor complexes (Figure 1A). Disheveled protein (DVL) is recruited by FZD, leading to LRP5/6 phosphorylation, Axin recruitment, and release of β-catenin from the destruction complex. Consequently, β-catenin regulates the transcription of target genes after translocating to the nucleus, forming complexes with the T cell–specific transcription factor (TCF) family of transcription factors. In normal livers, Wnt ligands are secreted by central-vein endothelial cells and stimulate pericentral hepatocytes with high β-catenin activity.^5^ This specific pattern can be detected by immunostaining for glutamine synthetase (GS), a Wnt/β-catenin downstream target in normal human livers (Figure 1B). This high β-catenin activity in normal mouse livers was also visualized based on Tcf/lymphoid enhancer-binding factor (Lef)-1 transcription factor promoter–driven green fluorescence protein tagged with histone (H)-2B tag (Lef:H2B: GFP) (Figure 1B). In contrast, aberrant β-catenin activation occurs in subsets of HCC, as evidenced by GS^+^ tumors in patients and in mouse lesions positive for Tcf/Lef1 transcription factor promoter activity (Figure 1B). β-Catenin is essential for alcohol metabolism and transcriptionally regulates key alcohol-detoxification enzymes, including cytochrome P450 (CYP)-2E1 in pericentral hepatocytes.^6^ Long-term ethanol exposure can lead to dysregulation of the Wnt/β-catenin pathway in ALD.^7^ M macrovesicular steatosis, lobular inflammation, hepatocellular ballooning, and necrosis are most pronounced in the central portions of the hepatic lobules during ALD development.^8^ As shown in Figure 1C, Wnt ligands (Wnt5a, Wnt5b, Wnt7b), receptors (FZD4 and -5, LRP5 and -6), DVL1, and transcriptional regulators (TCF7, β_1_-catenin) were down-regulated, whereas receptors (FZD-1, -2, -3, -6, and -7), LEF1, Axin2, the transcriptional regulator TCF4, and three Wnt-inducible secreted proteins (WISP1 to -3/CCN4 to -6) were up-regulated in the livers of patients with alcoholic hepatitis (AH). Disruptions in β-catenin signaling are evident across all stages of ALD, contributing to dysregulated lipid metabolism, oxidative injury, chronic inflammation, excessive extracellular matrix (ECM) deposition, and oncogenic transformation. This review systematically examines the multifaceted roles of Wnt/β-catenin signaling in ALD—from its modulatory effects in hepatic steatosis to its contributions to inflammation, fibrogenesis, and hepatocarcinogenesis^9^—and discusses emerging therapeutic strategies targeting this pathway. Furthermore, the ways in which advances in multiomics and precision medicine promise to revolutionize early diagnosis and patient-specific treatment strategies are addressed.

**Figure 1:**
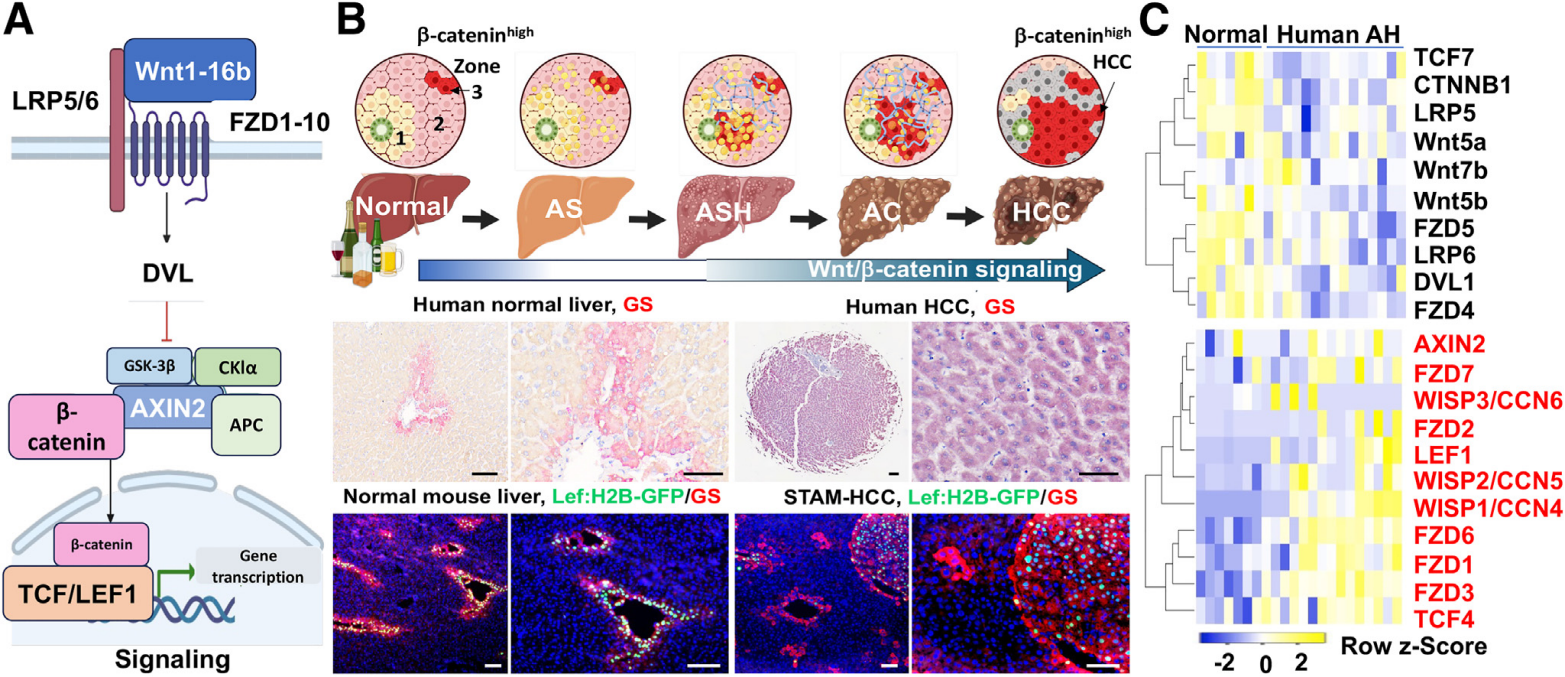
Altered Wnt/β-catenin signaling in alcohol-related liver disease (ALD). **A:** Schematic diagram showing the activation of the Wnt/b-catenin pathway. **B:** Diagram illustrating the progression of ALD to hepatocellular carcinoma (HCC). β-Catenin activity is normally high in hepatocytes within Zone 3 due to pericentral Wnt stimulation. Wnt/β-catenin signaling is decreased during alcoholic steatosis (AS) and alcoholic steatohepatitis (ASH). This pathway is up-regulated in fibrotic and tumorigenic responses during alcoholic cirrhosis (AC) and HCC. IHC analysis for glutamine synthetase (GS) on human normal and HCC livers is shown in the middle panel. Dual staining for GS (red signal) and green fluorescent protein (GFP; green signal) labels high β-catenin activity in normal livers and HCC induced by streptozotocin and high-fat diet in the Streptozotocin and High-Fat Diet–Induced NASH Model (STAM) (**bottom panel**). **C:** Heatmaps show up-regulated (labeled in red) and down-regulated genes of Wnt/β-catenin signaling in the livers of patients with AH compared to those in normal donors. Data were based on publicly available GDS4389 data set (*https://www.ncbi.nlm.nih.gov/gds*) the diagrams were generated using BioRender.com (Toronto, ON, Canada). The **arrow** delineates the path of liver disease evolution, from normal tissue through ALD to liver cancer. Scale bars = 200 mm.

## Steatosis and Wnt/β-Catenin Signaling

### Alcohol-Associated Steatosis

Alcoholic steatosis (AS) is the earliest manifestation of ALD, defined by an excessive accumulation of triglycerides in hepatocytes. Although steatosis is reversible, its persistence predisposes the liver to further injury and progression to steatohepatitis and HCC. Ethanol metabolism, primarily catalyzed by isozymes of alcohol dehydrogenase (ADH) and acetaldehyde dehydrogenase (ALDH), produces acetaldehyde and acetate. In parallel, the CYP2E1 enzyme generates reactive oxygen species (ROS), further contributing to hepatocellular injury. These toxic metabolites impair mitochondrial β-oxidation and stimulate *de novo* lipogenesis by activating sterol regulatory element-binding protein (SREBP)-1c.^10^ Moreover, alcohol-induced insulin resistance increases free fatty acid influx, while suppressed very low–density lipoprotein (VLDL) secretion hampers lipid export.^11^ These factors create a lipotoxic environment, marked by mitochondrial dysfunction due to oxidative stress, and endoplasmic reticulum stress, triggering inflammatory pathways like NF-κB.^12^

### Restoring Lipid Homeostasis

Reversal of fat accumulation within the liver depends on restoring the balance between lipid accumulation and clearance. Regulators like peroxisome proliferator-activated receptor (PPAR)-α and AMP-activated protein kinase (AMPK) promote fatty acid oxidation and suppress lipogenesis, reducing triglyceride buildup.^13^ However, alcohol exposure, compounded by genetic predispositions (eg, the E167K polymorphism in *TM6SF2*, a risk allele in alcoholic cirrhosis^14^), can disrupt these protective mechanisms, leading to persistent steatosis and a microenvironment favoring malignant transformation. Consequently, therapeutic strategies activating PPARα and AMPK—whether via pharmacologic agents or lifestyle interventions—are currently being explored to restore hepatic lipid balance.

### Role of Wnt/β-Catenin in Regulating Lipid Metabolism

The Wnt/β-catenin pathway is crucial for liver lipid metabolism, regulating transcription factors involved in lipogenesis and fatty acid oxidation. Under normal conditions, β-catenin regulates lipogenic transcription factors such as SREBP-1c and PPARγ.^15^ Activation of the Wnt/β-catenin pathway—especially through GSK 3β inhibition—up-regulates PPARα,^16^ enhancing fatty acid oxidation and promoting lipid clearance. However, alcohol seems to suppress Wnt/β-catenin signaling, as indicated by the down-regulation of downstream target genes during the AS stage.^17^ Deregulation of β-catenin likely results from its degradation via the HECT E3 ubiquitin ligase (HERC)-5–mediated ISG15 conjugation, which is up-regulated after alcohol exposure.^17^ Loss of β-catenin causes severe macrovesicular steatosis, increased liver injury markers, hepatic oxidative stress, and reduced superoxide dismutase (SOD)-2 expression, leading to systemic toxicity and early mortality.^6^ An Rs2302685 mutation in the gene encoding the LRP6 receptor has been associated with individual susceptibility to alcoholic liver injury related to the Wnt/β-catenin–TCF1–CYP2E1 signaling pathway.^18^ Autophagy and mitochondrial function exhibit distinct zonal regulation in the liver and are intimately linked to β-catenin signaling during steatosis progression. In zone 3 hepatocytes, impaired autophagic flux and mitochondrial dysfunction—exacerbated by ethanol metabolism—lead to lipid droplet accumulation and oxidative stress. β-Catenin, a key regulator of metabolic zonation, becomes aberrantly activated in this region under lipotoxic conditions, disrupting hepatocyte differentiation and promoting survival pathways. This convergence of defective organelle homeostasis and Wnt/β-catenin–driven transcription creates a permissive niche for steatotic injury and potential tumorigenesis. Pharmacologic restoration of Wnt/β-catenin signaling can reduce ALD progression *in vivo*.^19^ This protective role positions β-catenin as a promising therapeutic target for mitigating alcohol-induced steatosis and for preventing its progression to steatohepatitis and HCC.

### Crosstalk between Oxidative Stress and β-Catenin

Excessive alcohol consumption generates high levels of ROS, which overwhelm the antioxidant defenses of the liver, including SODs and catalase. Long-term ethanol intake depletes glutathione (GSH) and compromises mitochondrial integrity, further increasing ROS production. This oxidative environment damages lipids, proteins, and nucleic acids; impairs DNA-repair mechanisms; and triggers epigenetic alterations that predispose hepatocytes to oncogenic transformation. Interestingly, ROS can inhibit GSK-3β,^20^ resulting in the stabilization of β-catenin and the transcriptional activation of antioxidant genes such as *SOD2* and *CAT*. Reactivating Wnt/β-catenin signaling in experimental models of ALD has been found to reduce oxidative stress.^17^^,^^19^ Although this response may initially protect hepatocytes from oxidative damage, persistent β-catenin activation in the context of chronic oxidative stress may permit the survival of damaged cells, facilitating the accumulation of mutations and promoting carcinogenesis.^9^

### Steatohepatitis and Contextual Dependent Regulation of Wnt/β-Catenin Signaling

#### Inflammatory Cascades in ALD

The transition from simple steatosis to steatohepatitis involves a strong inflammatory response triggered by damage-associated molecular patterns from alcohol-induced hepatotoxicity. Additionally, gut-derived pathogen–associated molecular patterns contribute to liver inflammation due to the negative effect of ethanol on gut integrity. The immune cells of the liver, including Kupffer cells and hepatic stellate cells (HSCs), maintain homeostasis but induce chronic inflammation under ethanol exposure. Alcohol-induced gut dysbiosis increases intestinal permeability, allowing bacterial endotoxins like lipopolysaccharides (LPSs) to enter the liver. LPSs activate Kupffer cells via Toll-like receptors (TLRs), leading to the secretion of proinflammatory cytokines, such as tumor necrosis factor (TNF)-α, IL-1β, and IL-6, exacerbating hepatocyte damage and inflammation. In addition, alcohol can cause oxidative and endoplasmic reticulum stress, accumulating misfolded proteins and damaged organelles.^21^ These stress signals activate inflammasomes, such as NLRP3 (NOD-, LRR-, and pyrin domain-containing protein 3), within Kupffer cells, processing and releasing IL-1β and IL-18.^22^ Under physiological conditions, anti-inflammatory cytokines and regulatory proteins such as suppressors of cytokine signaling (SOCSs) counterbalance inflammatory responses; however, long-term alcohol consumption disrupts these mechanisms, leading to sustained inflammation.

Monocytes and neutrophils are quickly recruited to the liver due to increased concentrations of inflammatory cytokines and LPSs. Neutrophils release ROS, neutrophil extracellular traps (NETs), and proteolytic enzymes like neutrophil elastase, further exacerbating hepatocellular injury. Moreover, chronic inflammation can skew neutrophils toward a protumorigenic phenotype (N2), characterized by the secretion of cytokines such as IL-8^23^ and vascular endothelial growth factor (VEGF).^24^ This secretion creates an immunosuppressive microenvironment that facilitates HCC initiation and progression. Emerging biomarkers such as the neutrophil-to-lymphocyte ratio have shown promise in correlating with disease severity and survival outcomes in ALD and HCC.^25^

#### Wnt/β-Catenin in Inflammatory Regulation

Zonation-dependent hepatic inflammation in ALD involves spatially restricted β-catenin signaling, which modulates the expression of immune mediators across liver lobules, shaping regional susceptibility to injury and regeneration. Within this intricate network, the Wnt/β-catenin signaling pathway exerts dual and context-dependent effects on inflammation. Noncanonical Wnt ligands such as Wnt5a can activate NF-κB^26^ and inflammasome pathways, thereby amplifying the proinflammatory activity of Kupffer cells.^27^ Conversely, the activation of β-catenin in hepatocytes promotes the expression of IL-10,^28^ which counteracts the inflammatory response. Heatmap analysis indicates decreased expression of Wnt ligands, receptors, and β_1_-catenin in AH patients, as evidenced by data from the publicly available data set GDS4389 (*https://www.ncbi.nlm.nih.gov/gds/4389*, last accessed May 20, 2025) (Figure 1C). Thus, it can be postulated that the balance of pro- and anti-inflammatory modulation by Wnt/β-catenin signaling is disturbed in AH: β-Catenin signaling is down-regulated in hepatocytes while being up-regulated in immune cells, thus tipping the overall balance toward sustained inflammation for HCC development. Indeed, neutrophils contribute to liver inflammation and injury in ALD, and their activation is influenced by the Wnt/β-catenin pathway.^7^ Moreover, macrophages are a source and recipient of Wnt signals during tissue repair.^29^ Infiltrating macrophages are associated with steatosis-induced Wnt expression and promote the growth of tumor progenitor cells in obese patients.^30^ In mice, oncogenic β-catenin initiates an inflammatory reaction that influences the aggressiveness of HCC.^31^ Targeted modulation of this Wnt/β-catenin pathway to restore the equilibrium between pro- and anti-inflammatory signals represents a promising therapeutic avenue for mitigating liver injury and preventing HCC.

### Profibrogenic Action of Wnt/β-Catenin Signaling in Alcoholic Fibrosis and Cirrhosis

#### Pathogenesis of Fibrosis and Cirrhosis in ALD

Liver fibrosis pathophysiology is characterized by prolonged HSC and myofibroblast activation, leading to an overproduction of ECM components, notably fibrillar collagens. The complex interplay among hepatocytes, HSCs, portal fibroblasts, and resident macrophages—including Kupffer cells—elicits profibrotic cytokine secretion,^32^ which is amplified by paracrine and autocrine signaling loops for sustained liver fibrosis. Moreover, alcohol-induced gut dysbiosis facilitates the translocation of bacterial endotoxins into the portal circulation, exacerbating Kupffer cell activation and subsequent fibrogenesis. These interdependent mechanisms disrupt the balance between profibrogenic and antifibrogenic signals,^32^ promoting ECM deposition, architectural distortion of the liver, and impaired liver function.^33^ Advanced fibrosis is defined as F ≥ 2, and cirrhosis is defined as F ≥ 4 based on the METAVIR^34^ classification. Variations in *PNPLA3*, *MBOAT7*, and *TM6SF2* loci confer a risk for alcohol-related cirrhosis.^14^ As fibrosis advances to cirrhosis, extensive ECM deposition disrupts regular vascular and lobular organization, impairing blood flow and nutrient exchange.^35^ This impairment accelerates the loss of hepatocytes and activates compensatory regenerative pathways in the remaining hepatocytes, triggering DNA synthesis. During this process, the genome becomes more vulnerable to random mutations, some of which may drive neoplastic transformation and eventually lead to HCC. This altered hepatic microenvironment—characterized by chronic inflammation, oxidative stress, and genotoxic insults^36^—further promotes malignancy progression. As a result, fibrotic and cirrhotic livers are significantly more prone to HCC, highlighting the crucial importance of early intervention before fibrotic scarring becomes irreversible.

#### HSC Activation and TGF-β Signaling

A hallmark of ALD is the activation of HSCs. Under normal conditions, HSCs store vitamin A in lipid droplets. However, long-term exposure to ethanol and its toxic metabolite acetaldehyde triggers their transdifferentiation into myofibroblast-like cells.^37^ This phenotypic transformation is primarily driven by fibrogenic cytokines, notably transforming growth factor (TGF)-β, which binds to its receptors on HSCs and activates SMAD-dependent signaling cascade. The outcome is the increased expression of profibrotic genes that code for collagen (*COL1A1*, *COL1A2*), α-smooth muscle actin (*ACTA2*), and tissue inhibitors of metalloproteinases (*TIMPs*).^38^ Additional profibrotic mediators include platelet-derived growth factor (PDGF)^39^ and connective tissue growth factor (CTGF), which further sustain the fibrogenic process. CTGF is a matricellular protein and promotes cell adhesion via binding to cell surface receptors (ie, integrin, heparan sulfate proteoglycan) and extracellular proteins (ie, fibronectin, von Willebrand factor).^40^ It can directly bind to profibrotic ligands (ie, TGF-β, PDGF-B) and facilitate their presentation to cognate receptors, leading to persistent signalings.^41^ The Hippo effectors Yes-associated protein (YAP) and transcriptional coactivator with PDZ-binding motif (TAZ) are other key regulators of HSC activation.^42^ YAP/TAZ proteins are usually sequestered in cytoplasm via phosphorylation by Hippo kinases. Increased liver stiffness and altered cell–cell interaction in progressive liver fibrosis can activate YAP/TAZ.^43^ YAP/TAZ interacts with TGF-β signaling to amplify collagen synthesis.^44^ TGF-β and YAP can synergistically up-regulate CTGF in alcohol-accelerated and carbon tetrachloride–induced liver fibrosis.^45^ Furthermore, ALDH2 protein deficiency—a common finding in patients with alcohol-induced liver injury—exacerbates oxidative stress and promotes fibrotic signaling.^46^ ALDH2-deficient hepatocytes with TAZ activation produce harmful oxidized mitochondrial DNA via extracellular vesicles that contain CTGF, which promotes oncogenic pathways and HCC.^42^

#### Decline of Antifibrotic Regulation in ALD

The inherent antifibrogenic mechanisms of the liver, including TGF-β activation regulation, counteract these profibrogenic processes. This master regulator of liver fibrosis is normally secreted as an inactivated precursor form termed latent (L) TGF-β. The latency of deposited L-TGF-β is controlled by ECM1 in the healthy liver given that germline deletion of the *ECM1* gene causes spontaneous liver fibrosis with hyperactivation of the TGF-β signaling in mice aged 8 to 12 weeks.^47^ It was found that ECM1 binds to and sequesters CTGF from binding to a TGF-β activator-integrin αvβ6.^48^ This antifibrotic molecule is down-regulated in the liver of patients with alcoholic fibrosis and cirrhosis. In contrast, in mice, ectopic expression of this factor alleviates alcoholic fibrosis induced after Western diet feeding in combination with 10% to 20% alcohol administration.^48^

Another important aspect of the pro- and antifibrotic imbalance involves epigenetic and transcriptional reprogramming of liver cells. Prolonged exposure to the toxic byproducts of ethanol metabolism can lead to histone modifications and changes in DNA methylation that predispose HSCs to persistent activation.^49^ Concurrently, miRNA networks are altered: miR-29, which inhibits *ECM* gene expression, is frequently down-regulated in fibrotic livers, while certain miRNAs that promote fibrotic gene expression may be up-regulated.^50^ Transcriptional hepatocyte NF (HNF)-4a deregulation is a hallmark in AH. HNF4a is vital in maintaining hepatocyte differentiation and regulating gene expression in liver metabolism. In ALD, long-term alcohol consumption down-regulates HNF4α in hepatocytes, leading to impaired liver metabolic function.^51^ TGF-β promotes hepatic apoptosis, liver fibrosis, and suppression of hepatocyte proliferation during ALD development.^52^ This factor controls upstream transcriptome regulations and causes defective metabolic and synthetic functions by inducing the switch from the P2 to P1 promoter of HNF4α in hepatocytes.^53^ Increased expression of HNF4α transcribed by the P2 promoter indicates a poor prognosis in HCC.^54^ Forced re-expression of Hnf4α in cirrhotic rat livers resets target genes and reverses impaired hepatic function.^55^

Liver fibrolysis is a fibrosis resolution process involving ECM-degrading enzymes known as matrix metalloproteinases (MMPs). MMPs—including MMP-2, MMP-9, and MMP-13—can degrade collagen and other ECM proteins, attenuating fibrosis.^56^ However, in the context of ALD, the production and activity of MMPs are compromised, partly due to the overexpression of TIMPs. Even one instance of alcohol intoxication in teenagers can cause an imbalance in fibrosis markers, resulting in increased levels of TIMP1 and MMP-9 in the bloodstream.^57^ TIMPs—particularly TIMP1 and TIMP2—shift the balance toward ECM accumulation by binding to and neutralizing MMPs. Thick collagen fibrils deposited in the liver increase stiffness, alter fluid mechanics, and disrupt normal liver cell–cell communications, leading to sustained fibrotic reaction and, eventually, cirrhosis.

#### Wnt/β-Catenin Signaling in Fibrogenesis

Wnt/β-catenin signaling contributes to the activation of HSCs and the advancement of liver fibrosis, leading to cirrhosis. Canonical (Wnt3a and -10b) and noncanonical (Wnt4 and -5a) ligands, Fz1 and -2, and co-receptor LRP6 are induced in activated HSC.^58^ In human liver, Wnt signaling enhances their activation and survival.^59^ β-Catenin is overexpressed in liver fibrosis, and down-regulation of the Wnt/β-catenin signaling pathway inhibits HSC activation.^60^ In addition, Wnt3a and -5a significantly enhance HSC activation and fibrogenic response, particularly following liver injury, by promoting β-catenin–dependent transcriptional activity.^61^ Aurora kinase A (AURKA) has been shown to facilitate HSC activation and liver fibrosis through the Wnt/β-catenin pathway, suggesting a potential therapeutic target for fibrosis management.^62^ Schisandrin B has been identified as an inhibitor of HSC activation, which operates through modulation of the Wnt pathway, promoting macrophage phenotypic changes that contribute to HSC ferroptosis.^63^ Targeting of *WNT10A* by miR-378a-3p has been found to suppress HSC activation.^64^ The Wnt/β-catenin pathway is found to be activated by miR-17-5p to result in HSC activation through inhibiting Wnt inhibitory factor 1 expression.^65^ Additionally, noncanonical Wnt target genes such as *WISP1/CCN4* (Wnt-1–induced secreted protein 1, also termed *CCN4*) are induced by alcohol and contribute to HCC development.^66^ In ALD, β-catenin–driven signaling in pericentral hepatocytes modulates fibrogenic cascades in a zonation-specific manner, potentially influencing HSC activation and ECM remodeling predominantly within zone 3. A heatmap indicated its high induction in human AH livers (Figure 1C). WISP1/CCN4 is profibrotic and activates HSC in metabolic dysfunction-associated steatohepatitis and carbon tetrachloride-induced liver fibrosis.^67^ It is plausible that Wnt signaling through these Wnt-inducible matricellular proteins activates HSC and promotes fibrosis in AH.

Wnt signaling also mediates fibrosis through crosstalk with other pathways. For example, it can interact with TGF-β in regulating HSC activation, which is inhibited by the doublecortin domain containing 2 (*DCDC2*).^68^ The interplay between the Wnt/β-catenin and YAP/TAZ signaling pathways has also been shown to promote congenital hepatic fibrosis.^69^ Collectively, Wnt/β-catenin, TGF-β, and YAP/TAZ signaling converge in an intricate network governing the activation and maintenance of HSCs.

### Protumorigenic Action of Wnt/β-Catenin Signaling in Alcohol-Related Hepatocarcinogenesis

#### Cellular Stress Responses and Oncogenic Transformation

Long-term alcohol consumption triggers multiple intracellular stress responses—including necrosis, apoptosis, senescence, autophagy, and ferroptosis—that collectively determine hepatocyte fate. In the acute phase of ethanol toxicity, hepatocyte necrosis predominates due to intense metabolic stress, oxidative damage, and membrane rupture, releasing intracellular contents that provoke robust inflammatory response. While apoptosis serves as a controlled mechanism for eliminating damaged cells, long-term alcohol exposure disrupts mitochondrial integrity by increasing ROS production and depleting antioxidants such as GSH,^70^ thereby activating the intrinsic apoptotic pathway mediated by the B-cell lymphoma 2 (Bcl-2) family and caspases. When apoptotic capacity is overwhelmed by repeated injury, surviving hepatocytes may accumulate genetic mutations that predispose them to oncogenesis.^2^

In response to chronic injury, hepatocytes may enter a state of senescence, which initially serves as a tumor suppressor by halting fibrogenic response.^71^ However, senescent cells secrete a senescence-associated secretory phenotype (SASP) rich in proinflammatory cytokines and growth factors that, over time, can promote fibrogenesis and facilitate tumor progression.^72^ Similarly, autophagy works to clear damaged proteins and organelles in normally functioning cells; yet, long-term alcohol intake impairs autophagy, leading to the accumulation of cellular damage and increased inflammation. In later stages, tumor cells may exploit autophagy as a survival mechanism under metabolic stress. Ferroptosis, a form of controlled cell death that is influenced by the peroxidation of lipids dependent on iron, is also observed in ALD.^73^ Although ferroptosis can eliminate severely damaged cells, sublethal activation may promote genomic instability,^74^ further contributing to oncogenic transformation.

#### Wnt/β-Catenin in Cell Fate Modulation

Aberrant Wnt/β-catenin signaling is central to the oncogenic process in ALD. Ethanol-induced hepatocyte damage inhibits proliferation by suppressing the Wnt pathway, thereby blocking liver regeneration.^75^ Concurrently, Wnt signaling modulates apoptosis by down-regulating proapoptotic proteins [eg, Bcl-2–associated X protein (BAX)] and up-regulating antiapoptotic proteins (eg, BCL-XL), thereby promoting cell survival.^76^ However, prolonged activation of β-catenin induces p21-mediated senescence,^77^ impairs regenerative capacity, and fosters chronic inflammation. Moreover, Wnt signaling can inhibit autophagy, preventing the clearance of damaged mitochondria and exacerbating oxidative stress. These interrelated effects create an environment where genetically compromised hepatocytes can survive and accumulate oncogenic mutations, ultimately driving hepatocarcinogenesis.

#### Oncogenic β-Catenin Activation and Genetic Predisposition

β-Catenin–driven *CTNNB1*-mutated tumor represents 30% of human HCCs and is characterized by a specific transcriptomic profile with overexpression of classic β-catenin targets, such as GS.^78^ Alcohol-induced β-catenin activation within pericentral zones may foster a protumorigenic niche where disrupted zonation patterns favor clonal expansion and malignant transformation of hepatocytes, contributing to HCC emergence in ALD. Long-term ethanol consumption results in sustained activation of β-catenin and its downstream targets, including *WNT7A*, *GS*, *CCND1*, and *WISP1*/*CCN4*.^66^ These effectors drive hepatocyte proliferation and contribute to forming a protumorigenic microenvironment. β-Catenin also functions as an upstream regulator of specific miRNAs, such as miR-22-3p, that promote HCC stemness and metastasis by targeting *TET2*, a crucial regulator of DNA methylation.^79^ In addition, genetic studies have reinforced the oncogenic role of dysregulated Wnt/β-catenin signaling in alcohol-related HCC.^80^ Genome-wide association studies have identified mutations in the *WNT3A*-*WNT9A* locus as potential early triggers of β-catenin dysfunction.^81^ Furthermore, variants in genes such as *PNPLA3* and *WNT9A* have been linked to increased HCC susceptibility, whereas polymorphisms in *HSD17B13* are associated with a reduced risk for HCC and improved patient survival outcomes.^81^ The complex interplay between genetic predisposition, chronic inflammation, fibrosis, and aberrant β-catenin signaling creates a pathologic microenvironment that promotes malignant transformation.

### Therapeutic Strategies and Future Perspectives

#### Targeting the Wnt/β-Catenin Pathway

Given its central role in the pathogenesis of ALD, the Wnt/β-catenin pathway represents a promising therapeutic target.^82^ Several strategies for modulating this pathway are being investigated. Small-molecule inhibitors, biologics, and natural compounds that directly target β-catenin or its upstream regulators (such as GSK-3β inhibitors)^83^ have shown promise in preclinical studies. For instance, pharmacologic inhibition of GSK-3β has been demonstrated to stabilize β-catenin and promote PPARα-mediated fatty acid oxidation, thereby ameliorating hepatic steatosis.^84^ Likewise, antagonists such as Dickkopf-1 (DKK1)^58^ block Wnt signaling in HSCs, reducing ECM deposition and attenuating fibrosis.^85^ The inhibitor of cAMP-response element binding protein/β-catenin PRI-724 has demonstrated the ability to suppress HSC activation and encourage the resolution of inflammation mediated by macrophages.^86^ This natural compound, cordycepin, alleviates diabetes-associated liver fibrosis by inhibiting Wnt/β-catenin signaling, reducing HSC activation.^87^ These tools hold promising potential for the treatment of liver cirrhosis.

#### Combination Therapeutic Approaches

Due to the multifactorial nature of ALD, combination therapies are likely to be the most effective. Agents that restore redox balance—for example, *N*-acetylcysteine or novel antioxidants—can be combined with antifibrotic compounds (eg, galectin-3 inhibitors or TGF-β antagonists) to reduce ongoing liver injury and promote tissue repair.^88^ Additionally, immunotherapeutic strategies that were originally developed for viral hepatitis and nonalcoholic steatohepatitis are being adapted for ALD, particularly in patients with concurrent metabolic syndrome or viral co-infections.^88^ An integrated approach that combines antiviral, metabolic, and anti-inflammatory therapies within a personalized treatment regimen may offer the most significant clinical benefit.

#### Advances in Precision Medicine and Biomarker Development

The advent of precision medicine is revolutionizing the management of ALD. Advanced techniques such as single-cell RNA sequencing, high-resolution imaging, and integrated multiomics profiling allow for detailed characterization of the hepatic cellular landscape and the molecular pathways driving disease progression. These technologies also enable the identification of novel therapeutic targets and facilitate risk-based patient stratification. Emerging biomarkers—including circulating extracellular vesicles and specific miRNA signatures^89^—are proving beneficial for the early detection of subclinical liver injury, enabling timely intervention. Shortly, patients with distinct epigenetic or miRNA profiles may be candidates for targeted therapies that could prevent the need for liver transplantation.

## Conclusion

While the central mechanisms driving ALD progression—namely oxidative injury, immune dysregulation, and fibrogenic remodeling—are well documented, the future lies in the development of nuanced, patient-specific interventions. The Wnt/β-catenin signaling pathway is crucial in regulating these processes, which makes it a promising target for therapy. As the understanding of this pathway deepens, the integration of targeted Wnt modulators with strategies to reduce oxidative stress and modulate immune responses may ultimately enable the halt or reversal of the progression of ALD. Such advances might significantly reduce the incidence and burden of alcohol-related liver cancer, ushering in a new era of personalized, precision medicine for patients with ALD.

## Disclosure Statement

None declared.
